# X-ray Vision, or Induced Supernormal Sight

**Published:** 1903-03-15

**Authors:** J. A. Smalley


### "X-RAY VISION, OR INDUCED SUPERNORMAL SIGHT."

BY J. A. SMALLEY, D.D.S., M.D.

"There are more things in heaven and earth, Horatio, than are dreamt of in your philosophy."

"To understand the true philosophy of mind is one of the highest and most noble objects that can possibly engage the attention of any human being."

The chief purpose of this paper is to give a brief statement regarding the manifestation of what has been termed "X-Ray Vision or Supernormal Sight" by Leo Brett, the fifteen-year-old son of F. M. Brett, M.D., formerly Professor of Bacteriology and Physical Diagnosis in the College of Physicians and Surgeons, Boston, Mass.

Professor Brett's published statement is to the effect that while experimenting with hypnosis upon Leo when he was nine years old he succeeded in placing him in catalepsy, and upon sudden release from that state he exclaimed: "Oh, papa, I can see your bones!" This, he states, was a great surprise to him, and almost beyond his credence, but he proceeded to examine into the matter and tested his statement by asking him to describe to him what he saw. Professor Brett's statement is as follows: "He not only described my bones, but also described a Colles' fracture received when I myself was a little boy. Since that time he has been repeatedly hypnotized for similar examinations, and every statement which he has made has proved true by clinical experience, operation, or post-mortem examination. His appearance is in no way changed while examining. To even a close observer nothing in the slightest degree is noticeable in action or expression, except that there is certain intensity of attention while busy examining. His eyes are open, and he is conscious of all that is going on about him, and remembers what he sees as he would at any other time. His appearance is one of intense concentration, and after a short time he complains of weariness, but no more so than any work of equal attention would produce."

Leo's description of this supernormal sight is that "about the person being examined is a smoky red background, and through it there is flashing in every direction a pale greenlight similar to that seen in a Crookes tube. This light illuminates the patient so that any and all the internal organs are visible to his naked eye, together with their contents and motion." Professor Brett states that this light is best visible to Leo in what to others is absolute darkness, and then it tires him less to work, as it requires less energy on his part to exclude ordinary light—the light by which we ourselves see.

Having seen a statement of Leo's supernormal sight, I immediately wrote to Professor Brett requesting permission to visit him and his son, which request was cordially granted. Neither father nor son had previous knowledge of my existence.

Upon my arrival the Professor introduced his son to me and said, "Leo, Dr. S. wants a demonstration of your peculiar sight." The Professor then passed his hand before the young man's eyes, after having requested him to close them, and then said, "Now you can not open your eyes;" then, after passing his hand once more before his eyes, said, "Now you can open them and see."

Leo stood about six feet from me. I was standing in the full sunlight. I asked Leo if he could see my bones. He replied that he could. I then asked if he could tell me if the bones of my arms had ever been injured. He glanced quickly up and down my arms, and much to my surprise I felt a wave like motion, and by watching his eyes I discovered that the wave passed along with his direct line of vision, but extended no farther. I felt this motion only while his glance swept up and down rapidly. His father said, "Search carefully, Leo," and then he looked steadily at my left shoulder, and, as if searching very carefully, he proceeded slowly to examine the arm to the finger tips. Then he began at the right shoulder and proceeded in the same way until he came to the beginning of the lower third of my forearm, where his eyes rested for a moment, and then he said, "Your arm has been broken; the outside bone has been broken in two places, close together; there are two small ridges there, where the bone has united." He then placed his fingers upon his own arm to show me the exact place, and he was correct, as that accident had happened to me many years ago.

Leo Brett's supernormal sight surpasses the X-ray machine, inasmuch as he can see the valves of the heart performing their functions and the conditions of their surfaces, the diseased condition of nerve tracts, and any abnormal condition of the internal structures, not excepting the brain and meninges.

"When genious leads the way, and a new discovery is made," says Flammarion, "it is but natural that people in general should find themselves left behind; they can not understand the ways of progress.

"The discovery of the Roentgen rays, so inconceivable and so strange in its origin, ought to convince us how very small is the field of our usual observations. To see through opaque subtances! to look inside a closed box! to see the bones of an arm, a leg, a body, through flesh and clothes! Such a discovery is, to say the least, quite contrary to everything we have been used to consider certainty. This is indeed a most eloquent example in favor of the axiom: "It is unscientific to assert that realities are stopped by the limit of our knowledge and observation."

"We are told of five doors to human knowledge—sight, hearing, smell, touch and taste. These five doors open for us but a little way to any knowledge of the world around us, especially the last three—smell, taste and touch. The eye and ear can do a good deal, but it is light alone that really puts us in communication with the universe.'"

"Now what is light? It is caused by a kind of excessively rapid vibration of the air. A sensation of light is produced on our retina by vibrations which extend from 400 trillions a second (the red extremity of the luminous spectrum) to 756 trillions.

"*They have long ago been measured with precision*, and below and above these numbers are vibrations of ether not perceptible to our vision. Beyond the red line are dark caloric vibrations. Beyond the violet line are chemical vibrations, actinitic, and capable of being photographed, but all obscure."

"There are others still unknown to us. A comparison made recently by Sir William Crookes of the probable correspondence between these phenomena of the universe and the vacancies that our terrestrial organization seems to suffer from this continuity. Take a pendulum beating each second in the air. If we double its beats we obtain the following series of vibrations:

| Degree. | | |
|---|---|---|
| 1 | 2 | |
| 2 | 4 | |
| 3 | 8 | |
| 4 | 16 | |
| 5 | 32 | |
| 6 | 64 | |
| 7 | 128 | |
| 8 | 256 | Sound. |
| 9 | 512 | |
| 10 | 1,024 | |
| 15 | 32,768 | |
| 20 | 1,047,576 | Unknown. |
| 25 | 33,554,432 | |
| 30 | 1,073,741,824 | Electricity. |
| 35 | 34,359,738,368 | |
| 40 | 1,099,511,627,776 | Unknown. |
| 45 | 35,184,372,088,832 | |
| 48 | 281,474,976,716,656 | |
| 49 | 562,949,953,421,312 | Light. |
| 50 | 1,125,890,906,842,624 | |
| 55 | 36,028,797,018,963,268 | |
| 56 | 72,057,594,037,927,936 | Unknown. |
| 57 | 144,115,188,075,855,872 | |
| 58 | 288,230,376,151,711,744 | |
| 59 | 576,460,752,303,423,488 | |
| 60 | 1,152,921,504,606,846,976 | X-rays. |
| 61 | 5,305,843,009,213,693,952 | |
| 62 | 4,611,686,018,427,389,904 | Unknown."[1] |
| 63 | 9,223,372,636,854,775,808 | |

Mr. A. Pizon has shown[2] "that the pigmentary granules that accompany the visual cells are, without exception, in rapid motion. The presence of these granules in immediate contact with the visual cells and the constancy of their motions leads naturally to the conclusion that they serve as intermediaries in the excitation of the cells. The granules acquire their energy from the light and transmit it to the rods and cones of the retina with which they are in contact, and from them it travels along the optic nerve."

Normal sight then depends upon the pigmentary granules being sufficiently sensitive to respond to vibrations of from 400 trillions to 756 trillions per second. Vibrations of this rapidity will not pass through what we call opaque substances, for the reason that the wave lengths of such vibrations are broken up, the spaces between the atoms composing such

[1] "The Unknown," by Camille Flammarion.
[2] See *Dental Brief*, June, 1892.

substances either not being large enough, or the character of such atoms themselves changing the wave lengths.

The air is too heavy a body to respond to many of these vibrations, but, as the ether of space passes through the air and all bodies we have any knowledge of, these wonderfully rapid waves, like the X-rays, pass through many substances regarded as solid, such as wood, metals and our own tissues.

If by any means we can increase the sensitiveness of the nerves of sight or hearing or of tactile impressions, then we can have supernormal sight, supernormal hearing, supernormal feeling, or supernormal mental action, for the brain cells also are reached by these ether waves.

We know that in strychnin poisoning the tactile nerve fibres are made hyperæsthetic to such an extent that the slightest disturbance of the air will throw the patient into a clonic spasm.

Hypnotism produces supernormal responsiveness to the nerves of sight and hearing, to tactile impressions and mental action. Hypnotism is a sleep produced by confining the mental action of the individual to one idea in a monotonous and soothing way until the mind becomes exhausted and the patient sleeps.

The length of time required to establish the hypnotic influence varies with the subject; some persons require continuous effort for two or three hours, or may require an hour's effort repeated many times, or, in case the patient resists, it may be accomplished only after the systemic effects of morphin have been produced.[1]

The time required depends upon the idiosyncrasy of the individual and upon the number of impressions of this kind already made in the nerve tracts of the patient; for, like all impressions in nerve tracts, this faculty will become automatic in time, so that the individual may be able to bring this condition about by his own will, or the operator to produce the effect by a wave of the hand, a word or a glance of the eye, or in other words by suggestion.

[1] "Hypnotism in Mental and Moral Culture," by Professor J. Duncan Quackenbos, M.D.

Suggestion may mean much or little. To one who is on his guard it has no effect, but to one who is passive or supernormally sensitive it may mean a ruined life, ill health, insanity, or possibly sudden death. This fact shows us how incumbent it is upon medical and dental faculties to make the study of physchology and hypnotism a part of their college curricula, not only to develop these relatively new branches of science for the benefit of mankind, but also to guard the community against those ignorant and unprincipled charletans who do so much injury by their abuse of hypnotic power, and who by their fraudulent practices bring into contempt a science which, properly directed and in the hand of instructed, conscientious, and qualified operators, may be made a beneficent agency for the relief of human suffering.

Suggestion from one person to another is normally made by the vocal cords vibrating by an effort of the will, thus setting up waves in the atmosphere which vibrate within the range indicated by Professor Crookes' table. Within this range they act upon the tympanum malleus, incus, stapes, and auditory nerve and auditory centre in the medulla oblongata. Just where or how they take on the rapidity of movement necessary for their conversion into mentality is not known, but it must be beyond the auditory nerve and within the substance of the auditory centre, or, possibly, in the field of brain cells which perform their function as a result of sound wave stimuli. The motion of sound waves must be changed to a more rapid vibration than the normal ear receives, for the brain cells, in order to perform their function of producing mentality, certainly act under exceedingly high vibrations. Otherwise, they would not be capable of responding to the very vibrations of the X-ray.

Suggestion can be conveyed from one mind to another by other methods than by the use of the ear, eye, or sense of touch. I refer to supernormal mental action, such as telepathy, thought transference, or so-called mind-reading, which prove that the brain cells are capable of vibrating in unison with such waves as are too rapid to be carried by the air and can only be conveyed by the ether.

The brain cells are endowed with the power of sending out and receiving unspoken thoughts through the ether just as the vocal cords and the tympanum, or the telephone transmitter and receiver. do sound waves, or Marconi's wireless telegraph instruments transmit Hertz's electro-magnetic waves, this being but a further development of Faraday's discovery of the induction coil.

As an illustration and demonstration of the fact above stated, I will describe an experiment tried while with others visiting at the house of a friend. The experiment was new to us, but as a game, was introduced in London several years ago.

The hostess, Mrs. P., was blindfolded. We sat in a circle and joined hands. except where I used my right hand, with which I picked up a card from a pack which I had just shuffled. I held the card for all to see, excepting the lady who was blindfolded. She could not possibly see the card.

I requested her to name the first card which she either saw mentally or thought of. After about three minutes, during which time we all kept our minds upon the card I held, she replied, "I think of the five spot of spades," and this was the exact card I held. We then tried again; she got the idea of a red card, either a king or a queen. It was the king of hearts.

We then tried with my wife; she failed. We then tried with my little girl. She said that she could see a club. After waiting a while she could yet see but one club. The card was a six spot of clubs.

We then tried with Miss P., a school teacher and daughter of our hostess. She soon replied, "I see diamonds." I asked her how many. In a moment she replied, "Three." It was the three of diamonds I held.

Upon a second trial she named the character of the card, but did not have the number quite right. Upon a third trial she got the character of the card, but could not give the number; She said: "I can not make out the number; I get three and four." She then said, "You are not keeping your minds upon the card." I asked her how she knew, and she replied, "I feel it." This surprised me, for I was thinking it was hard to keep the ten spots impressed on my mind as they were on the card, four spots on each side and two in the middle, and these three unequal rows bothered me, and, as she had been slow to reply, I had tried to fix the mental image of a tobacco pipe on her mind. It was at this point she exclaimed, "You are not keeping your minds on the card."

On another evening we tried the same experiment upon Mrs. P. On this occasion she was quite slow to reply. I finally asked her if she could get no impression. She said, "Yes," then hesitated a moment and then said, "It is the king of spades," which was correct. She would not try the experiment again, as she said she was startled very much by the fact of seeing the card in all its details, as with the naked eye.

A person who is pyschologized is not necessarily asleep but is for the time being mentally hyperesthetic. The same principles apply to psychology or purely mental phenomena that apply to hypnotism—passiveness must exist in both cases.

To illustrate idiopathic supernormal hearing or clairaudience, I will recite an incident which occurred in my own family. One afternoon my wife permitted our little boy Paul to visit a playfellow who lived more than half a mile away. During the afternoon, while busy with her household duties, she told my mother that she heard Paul crying, and she rushed out of the house and searched the yard, barn and street, but, not seeing the child about, she returned to her work, only to drop it and make another search, declaring to my mother that she heard Paul crying pitiously and calling, "Mamma, mamma, mamma!" Again she failed to find him. After returning to her work she again stopped, saying she would go after Paul, as she still heard him crying. She went to the front gate, and, looking down the street quite a distance saw the lady at whose house the child had been visiting coming up the street leading Paul by the hand. His head was in bandages. She then learned that he had fallen down the cellar-way, and that he had cried a great deal and called for mamma. He was cut and bruised and badly frightened, but not seriously injured.

On another occasion my wife had idiopathic supernormal sight or clairvoyance which occurred in her sleep. She has had several minor cases of supernormal vision while awake, but this one will answer my purpose of illustration. One morning, when she was a child ten years of age, she came down stairs crying, and upon being asked by her mother what was the matter she replied that she had just had such a terrible dream, in which she had dreamed that she saw Mr. John King, who lived next door and was the father of her little playmate, all cut and bleeding and crawling on the ground, and that he was dying. Mr. John King was a travelling man, and that night he was coming home. He was in a berth in a sleeper, the door of which was unlocked. He evidently walked out of the car in his sleep and after falling off had crawled quite a little distance from the track, leaving a trail of blood.

On one occasion I had an idiopathic clairvoyance or supernormal vision. I was reading ancient history. I had been thus occupied for some time, and as the subject was very dry to me I read on in an uninterested way, when unconsciously I brought my left thumb up over the side of the book, and as it came into my field of vision I saw a cloth tied with a thread around the end extending to the first joint. I saw it but a moment, but very distinctly, and, knowing that it was an illusion, my mind was immediately aroused to activity. The vision at once vanished. The occurance was so singular that I looked at my watch to make note of the time, which was eleven o'clock, for reference in case anything should occur which would seem to be related with the phenomenon. At noon I went home to lunch, and as I turned up my plate my little girl, who had climbed up on her chair beside me, turned up her plate, and in doing so brought her left thumb into my field of vision, and I saw that it was tied with a cloth exactly as I had seen my own thumb an hour before. Upon questioning her mother I found that about eleven o'clock she had tied up the child's thumb on account of a slight cut. I asked how she knew the time. She replied that she had just looked at the clock to see if it was time to prepare luncheon.

Leo Brett says that physical contact, while in this supernormal condition, causes him more pain than does contact with a 110-volt electric current. Father Neri, who lived from 1515 to 1595 and who was a very enthusiastic and successful church advocate, had a normal heart-beat of 120, and the sound of his pulsations was audible to one standing near him. There is authentic evidence to show that his organism generated electro-magnetism of considerable power, but not at the instance of his will. There is also authentic evidence that D. D. Home (1863), whose physique was supernormal from birth, was capable of generating electro-magnetic force at will.

My attention was attracted to the subject of man's organism generating electric force by an incident which has happened to me but once to my knowledge. I was sitting quietly in my laboratory, when an unwise fly flew swiftly across the room and attempted to alight on my hair, and as he came in contact with the ends of some of the hair there was an electric discharge quite audible to me, and he fell to the floor, where he spun around on his back for a few moments and then flew away, apparently a much surprised fly; at least, I am sure he left a very much surprised witness to this phenomenon.

The electric fishes, narcine, thunderfish, and gymnotus, prove that it is possible for living organisms to generate and discharge electric force at will. There is only one combination of elements that possesses and supports life on this globe, and that is albumin, both vegetable and animal, and this not without heat, and not then unless the albumin is in a condition to generate electro-magnetism. Professor Loeb's experiments show that electro-magnetic force plays an important role in cell formation and cell function.

F. A. Messner, M.D., in 1778 advanced the theory that there was a force which caused motion, heat, light, electricity, magnetism, and life. Within the next seventy-five years, Grove, Helmholz, Mayer, Faraday, Liebig and Carpenter, by scientific demonstrations proved that motion, light, heat, electricity, and magnetism are co-related forces, and to this principle thus demonstrated the nineteenth century largely owes its great progress.

To-day science teaches us that not only motion, heat, light electricity and magnetism, but life and mind are co-related forces. What progress, especially in metaphysics, the twentieth century will owe to this fact is yet to be seen.

Science then teaches us that every atom of matter in the universe is imbued with that supreme force, the mind of Deity, and that this innate property of matter causes organized life, which is the one thing above all others that shows intelligence, chaos does not. Thus we see that the Christian religion has nothing to fear from the advancement of science, either in a physical or metaphysical direction.

The ark, Religion, has been tossing for centuries upon the waves of the black and stormy ocean of ignorance and superstition. Science is an adamant pier, rising slowly but surely above these waves which beat against it in vain, and on which this ark is destined to rest and form the completed beacon, from whose base it will receive increasing power, sending its radiance down the coming ages and lighting up all dark corners of the earth.—*Brief.*

